# Neutrophil-Derived Extracellular Vesicles Activate Platelets after Pneumolysin Exposure

**DOI:** 10.3390/cells10123581

**Published:** 2021-12-18

**Authors:** Eleftheria Letsiou, Luiz Gustavo Teixeira Alves, Matthias Felten, Timothy J. Mitchell, Holger C. Müller-Redetzky, Steven M. Dudek, Martin Witzenrath

**Affiliations:** 1 Division of Pulmonary, Critical Care, Sleep and Allergy, University of Illinois at Chicago, Chicago, IL 60612, USA; sdudek@uic.edu; 2 Division of Pulmonary Inflammation, Charité-Universitätsmedizin Berlin, Corporate Member of Freie Univeristät Berlin and Humboldt-Univeristät zu Berlin, 10117 Berlin, Germany; luizgustavo.ta@gmail.com (L.G.T.A.); Matthias.felten@charite.de (M.F.); holger.mueller-redetzky@charite.de (H.C.M.-R.); martin.witzenrath@charite.de (M.W.); 3 Department of Infectious Diseases and Respiratory Medicine, Charité-Universitätsmedizin Berlin, Corporate Member of Freie Univeristät Berlin and Humboldt-Univeristät zu Berlin, 10117 Berlin, Germany; 4 Institute of Microbiology and Infection, College of Medical and Dental Sciences, University of Birmingham, Birmingham B15 2TT, UK; t.j.mitchell@bham.ac.uk; 5 German Center for Lung Research (DZL), Justus-Liebig-University Giessen, 35390 Giessen, Germany

**Keywords:** microvesicles, pneumonia, NETs, *Streptococcus pneumoniae*, lung injury

## Abstract

Pneumolysin (PLY) is a pore-forming toxin of *Streptococcus pneumoniae* that contributes substantially to the inflammatory processes underlying pneumococcal pneumonia and lung injury. Host responses against *S. pneumoniae* are regulated in part by neutrophils and platelets, both individually and in cooperative interaction. Previous studies have shown that PLY can target both neutrophils and platelets, however, the mechanisms by which PLY directly affects these cells and alters their interactions are not completely understood. In this study, we characterize the effects of PLY on neutrophils and platelets and explore the mechanisms by which PLY may induce neutrophil–platelet interactions. In vitro studies demonstrated that PLY causes the formation of neutrophil extracellular traps (NETs) and the release of extracellular vesicles (EVs) from both human and murine neutrophils. In vivo, neutrophil EV (nEV) levels were increased in mice infected with *S. pneumoniae*. In platelets, treatment with PLY induced the cell surface expression of P-selectin (CD62P) and binding to annexin V and caused a significant release of platelet EVs (pl-EVs). Moreover, PLY-induced nEVs but not NETs promoted platelet activation. The pretreatment of nEVs with proteinase K inhibited platelet activation, indicating that the surface proteins of nEVs play a role in this process. Our findings demonstrate that PLY activates neutrophils and platelets to release EVs and support an important role for neutrophil EVs in modulating platelet functions in pneumococcal infections.

## 1. Introduction

*Streptococcus pneumoniae* (or pneumococcus) is a Gram-positive bacterium that represents a major causative pathogen of community-acquired pneumonia [1]. When pneumococcal lung infections are severe, they result in a series of events that include the dysregulation of the innate immune system and failure of the lung barrier [2]. These events cause invasive disease and life-threatening complications, with sepsis and acute respiratory distress syndrome (ARDS) being among the most important ones [2]. Despite antibiotic treatment and the availability of vaccines, pneumococcal pneumonia remains a leading cause of morbidity and mortality worldwide, and a better understanding of its pathobiology is needed. *Streptococcus pneumoniae* possesses a variety of virulence factors that contribute to disease pathogenesis [3]. Among these, pneumolysin (PLY), a pore-forming toxin released from bacteria, is expressed by almost all clinical isolates of *S. pneumoniae* and plays an essential role in the development and progression of pneumococcal pneumonia and lung injury [4]. PLY has a range of biological functions that contribute to key disease processes. These include direct cytotoxicity through cell lysis, the activation of multiple pro-inflammatory pathways, the promotion of extrapulmonary dissemination of *S. pneumoniae*, and the disruption of the immune system [5,6,7].

Neutrophils, which are key cellular components of the innate immune system, play a critical role in the host defense against *S. pneumoniae* [8]. Upon recognition of the invading bacteria, neutrophils are recruited to sites of injury and exert a range of potent antimicrobial activities. Despite this beneficial role, excessive neutrophil activation is associated with host injury and the aggravation of inflammation in pneumonia and lung injury [9]. Several neutrophil-derived products contribute to inflammatory processes. For example, in response to a variety of insults, neutrophils release neutrophil-extracellular traps (NETs), which are web-like structures comprised of DNA, histones, and neutrophil proteins (e.g., elastase, myeloperoxidase) [10]. Although NETs were initially recognized as a host defense mechanism to trap and eliminate bacteria, it became evident that they are also highly pathogenic, as they cause direct tissue damage and promote inflammation, coagulation, and other injurious responses [11]. NET levels increase significantly (in circulation and local inflamed tissues) in numerous pathological conditions, including pneumonia, sepsis, and acute lung injury syndromes (ALI) such as ARDS [12,13,14].

In addition to NETs, activated neutrophils release extracellular vesicles (EVs). EVs are a heterogeneous group of small membrane-enclosed vesicles released by cells into the extracellular space. The most well-studied types of EVs are exosomes (<~150 nm) and microvesicles (<~1 μm) [15,16]. These vesicles carry cargo that reflects the status of the cell of origin and play a critical role in intercellular communication by transferring molecular messages from one cell to another. Moreover, it is now well established that EVs generated under inflammatory conditions mediate disease pathogenesis and can also serve as biomarkers of disease. EVs derived from neutrophils (nEVs) are increased in patients with pneumonia, sepsis and ARDS [17,18,19]. Similar to NETs, nEVs can exert both antimicrobial and inflammatory activities [20,21]. 

Alongside neutrophils, platelets have been linked to pneumonia and ALI pathogenesis as they promote thrombosis, inflammation and regulate host defense mechanisms [22,23]. One mechanism by which activated platelets mediate their effects is through the release of EVs. Platelet-derived EVs (pl-EVs) are the most abundant EVs in the blood and have gained significant interest as inflammatory and pro-coagulant mediators and potential diagnostic markers of disease [24], including SARS-CoV-2 pneumonia [25]. Another mechanism by which platelets participate in inflammatory processes is through interaction with neutrophils [26]. Platelets and neutrophils modulate each other’s functions through several mechanisms. For example, activated platelets induce NET formation [27], while NETs from activated neutrophils induce platelet aggregation [28]. A few studies have also suggested a role for EVs in mediating platelet–neutrophil interactions [29,30], but the precise mechanisms of this interplay remain unclear. 

Both neutrophils and platelets are activated by *S. pneumoniae* and PLY [8,22], however, few studies have explored how these inflammatory stimuli alter the important interaction of both cell types. The present study aims to characterize the effects of PLY on neutrophils and platelets and explores the hypothesis that PLY-induced neutrophil extracellular products (EVs and NETs) contribute to platelet activation. Our data demonstrate that PLY is not only a potent stimulus for the release of neutrophil and platelet EVs, but it also triggers communication between neutrophils and platelets that involves EVs. 

## 2. Materials and Methods

### 2.1. Ethics

Blood for isolating neutrophils and platelets was obtained from healthy volunteers. All participants gave informed consent, and the procedure was approved by the local institutional review board—Ethics committee of the Charité-Universitaetsmedizin Berlin, Germany (EA4/032/17). Animal experiments were approved by institutional (Charité-Universitaetsmedizin Berlin) and governmental authorities (State Office for Health and Social Affairs (Landesamt für Gesundheit und Soziales) Berlin, Germany). 

### 2.2. Human Neutrophil Isolation

Peripheral blood from healthy volunteers was collected into 3.2% sodium citrate tubes. After collection, the blood was centrifuged to separate the platelet-rich plasma (PRP) from blood cells. Neutrophils were isolated as we have described before [7]. Cell viability was evaluated by trypan blue exclusion, and purity (>95%) was verified by flow cytometry (FACS). After isolation, neutrophils were resuspended in Hank’s balanced salt solution with Ca^2+^/Mg^2+^ (HBSS+/+), quantified, and used for experiments as indicated. 

### 2.3. Murine Neutrophil Isolation

Murine neutrophils were isolated from single-cell suspensions of bone marrow using the EasySepTm mouse neutrophil enrichment kit (Stemcell Technologies, Vancouver, BC, Canada). 

### 2.4. Neutrophil Treatments

To assess the activation status of neutrophils after pneumolysin (PLY) treatment, cell suspensions (0.5–1 × 10^6^ cells) were treated with PLY (100–500 ng/mL) for indicated times at 37 °C. PLY (~2.5 × 10^4^ HU/mg) was produced and purified as described elsewhere [31]. After treatment, cells were separated by centrifugation at 350 g for 5 min and processed for flow cytometry analysis. Cell-free supernatant was further centrifuged at 4000× *g* for 10 min. The final supernatant was stored at −80 °C until further use. 

### 2.5. Neutrophil Extracellular Trap (NET) Analysis and Isolation

The kinetic release of NETs was assessed using a SYTOX Green assay as described before, with some modifications [32,33]. Briefly, human or mouse neutrophils (1 × 10^5^ cells) were seeded onto 96-well black plates. The cell-impermeable DNA binding dye, SYTOX green, 5 μΜ, (ThermoFisher Scientific, Darmstadt, Germany) was added to each well and the cells were stimulated with PLY or HBSS as control (Ctr). Fluorescence was recorded for 4 h (15-min intervals) at 37 °C using a Spectramax M2 microplate reader (Molecular Devices). In a separate set of experiments, human neutrophils were pre-treated with U0126 (25 μM), a MEK inhibitor, and then treated with PLY. For each condition, the area under the curve was calculated. 

For NET visualization, human neutrophils (0.7 × 10^5^ cells in 200 μL HBSS+/+) were seeded in 8-well slides (Ibidi, Munich, Germany). Cells were allowed to attach, then treated with PLY, fixed in 3% PFA, and stained with DAPI. Images were taken at 40x magnification using an LSM 780 confocal laser-scanning microscope (Zeiss) and analyzed using the Zen 3.1 software (Zeiss). NET isolation was performed as described elsewhere [34], with some modifications. Briefly, human neutrophils (1–2 × 10^6^ cells) were allowed to attach in culture dishes and then treated with PLY. After 4 h, the cell supernatant was removed, and a solution of 0.5 U/mL micrococcal nuclease in HBSS+/+ (ThermoScientific, Darmstadt, Germany) was added carefully on the top of the cells. Following a 10 min incubation at 37 °C, the solution was collected, mixed with PBS (no calcium), and centrifuged at 300× *g* for 5 min to remove cells and debris. The supernatant (containing NETs) was stored at −80 °C until further use.

### 2.6. Neutrophil Elastase Assay

Elastase activity was measured using a colorimetric kinetic assay. Briefly, neutrophil supernatants or isolated NETs were mixed with elastase substrate (MeOSuc-Ala-Ala-Pro-Val-pNA, Calbiochem) in Tris 100 mM, pH 8. Absorbance was recorded at 37 °C for 30 min, and the ΔOD/min was calculated for each sample. 

### 2.7. Reactive Oxygen Species (ROS) Production

Human neutrophils (1 × 10^5^ cells) were seeded in a 96-well white plate, and then luminol reagents were added to each well (50 μM luminol and 1.2 U/mL HRP substrate). After 5 min, neutrophils were treated with PLY, and luminescence was recorded with the SpectraMax L (Molecular Devices) over a period of 30 min (1 min interval). The area under the curve was calculated for each condition.

### 2.8. Neutrophil Extracellular Vesicle (nEV) Isolation 

EVs were isolated from the cell-free supernatant of neutrophils after centrifugation at 20,500× *g* for 45 min at 4 °C, as we have described before [7]. EVs were washed, resuspended in PBS, and stored at −80 °C until further use. For a subset of experiments, nEVs were incubated with 50 μg/mL proteinase K (PanReac AppliChem, Renningen, Germany) for 60 min at 37 °C and washed in PBS before use. 

### 2.9. Platelet Treatments

Platelet-rich plasma (PRP) was diluted with Dulbecco’s phosphate-buffered saline with Ca^2+^/Mg^2+^ (DPBS+/+). Platelets (diluted PRP) were treated with PLY (vs PBS), neutrophil EVs or NETs (derived from Ctr or PLY-treated neutrophils; 2 × 10^6^ cells/mL) or EV-depleted supernatants at 37 °C. At the indicated times, the platelet suspension was diluted with PBS (no calcium) and was processed immediately for flow cytometry analysis.

### 2.10. Flow Cytometry 

All flow cytometry reagents were purchased from BioLegend (San Diego, CA, USA) unless specified. A BD FACS Canto II was used for all analyses. FACS data were analyzed using the FCS Express 6 Flow cytometry software (De Novo Software). Human neutrophils were analyzed by FACS after staining with CD62L-Alexa Fluor488, CD11b-Brilliant Violet 510, annexin V-APC, and 7-AAD. Neutrophil-derived EVs were analyzed after staining with CD66b-Pacific Blue (human nEVs) or Ly6G-FITC (murine nEVs) and annexin V-APC in HBSS+/+. Negative controls included staining of nEVs with corresponding isotype antibodies in buffer that does not contain calcium (HBSS−/−, EDTA). Platelet suspensions were stained with CD41-Alexa Fluor488 (or PE-Cy7 for murine platelets), CD62P-PercP 710 (eBioscience, San Diego, CA, USA), and annexin V-APC. EVs were gated based on size using 1 μm beads (Sigma-Aldrich). To ensure that the isolated EVs are vesicles and not cellular debris, annexin-V-stained EVs were incubated with 0.1% Triton X-100 (a detergent that dissolves membranes structures and disintegrates EVs) and then analyzed by FACS, as described [7,35,36]. For cell/EV quantifications, CountBright absolute counting beads (7 μm) were added in each sample, and calculations were performed according to the manufacturer’s instructions (Invitrogen, ThermoFisher, Darmstadt, Germany). All buffers used for EV and platelet analysis were double filtered (0.2 μm). 

### 2.11. Murine Model of S. pneumoniae Infection

Female C57BL/6 mice, 9–11 weeks old (Charles River, Germany) were anesthetized and inoculated intranasally with 5 × 10^6^ colony-forming units of *S. pneumoniae* serotype 3 (PN36), as described before [37,38]. Sham-infected mice (controls) received an equal volume of sterile PBS (controls). BAL (bronchoalveolar lavage) was obtained 48 h post-infection, by lavaging the lungs with 0.8 mL PBS containing cOmplete Mini protease inhibitors (Roche Diagnostics GmbH, Penzberg, Germany). The BAL fluid was centrifuged at 300× *g* for 10 min to separate the cells, and the supernatant (BAL fluid) was stored at −80 °C until analysis (BALF). BAL neutrophil counts were quantified by FACS as we have described previously [38]. BALF was further centrifuged at 2500× *g* for 10 min before EV isolation (as described above for nEVs) [7]. Blood was collected, and platelet counts were determined using a Scil Vet abc hematology analyzer. 

### 2.12. Statistical Analyses

GraphPad Prism 8 software was used for statistical analyses and graph preparation. T-test or one-way ANOVA (Tukey’s post hoc test) were used for comparisons of two or three groups, respectively. Non-parametric tests were used if data did not pass the normality test (indicated in figure legends). Experiments were performed at least 3 independent times. Values are expressed as means ± SEM. *p* values less than 0.05 were considered statistically significant.

## 3. Results

### 3.1. Neutrophil Activation upon Pneumolysin Treatment

**Figure 1:**
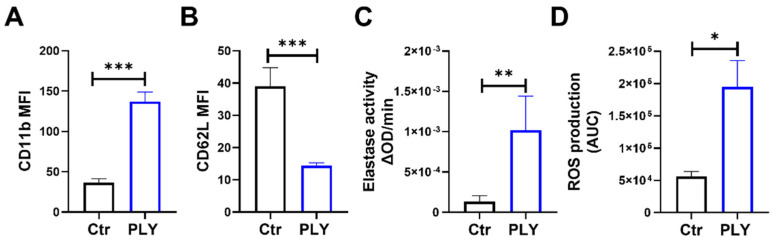
Pneumolysin (PLY) induces neutrophil activation. Human neutrophils were treated with PLY (100 ng/mL, 30 min) and cell surface expression (MFI) of (**A**) CD11b or (**B**) CD62L was assessed using flow cytometry. (**C**) Elastase activity was determined in cell culture supernatants. (**D**) ROS production was measured using a luminol based kinetic assay. Bars represent area under the curve (AUC) values calculated from kinetic curves. *n* = 5–9. * *p* < 0.05, ** *p* < 0.01, *** *p* < 0.001, *t*-test.

Upon activation, neutrophils undergo multiple changes that include cell surface protein alteration, the release of granule content, the production of reactive oxygen species (ROS), and the formation of NETs [39]. For our study, we initially assessed the activation status of isolated human neutrophils following pneumolysin (PLY) treatment. We monitored the cell surface expression levels of CD11b and CD62L in PLY-challenged cells by FACS, as CD62L shedding and CD11b-increased expression represent common neutrophil activation markers [40,41]. Figure 1A,B demonstrate that PLY stimulation resulted in a significant increase in expression of CD11b, and a decrease in expression of CD62L (CD62L expression decreases by shedding). Neutrophil elastase is stored within cytoplasmic azurophilic granules and released upon neutrophil activation. As expected, PLY treatment upregulated the secretion of elastase, as indicated by the increased levels of neutrophil elastase activity measured in the cell supernatants (Figure 1C). Furthermore, a potent induction of ROS generation was observed in PLY-treated neutrophils compared to the controls (Figure 1D). 

### 3.2. Pneumolysin Induces Neutrophil Extracellular Trap Formation 

**Figure 2:**
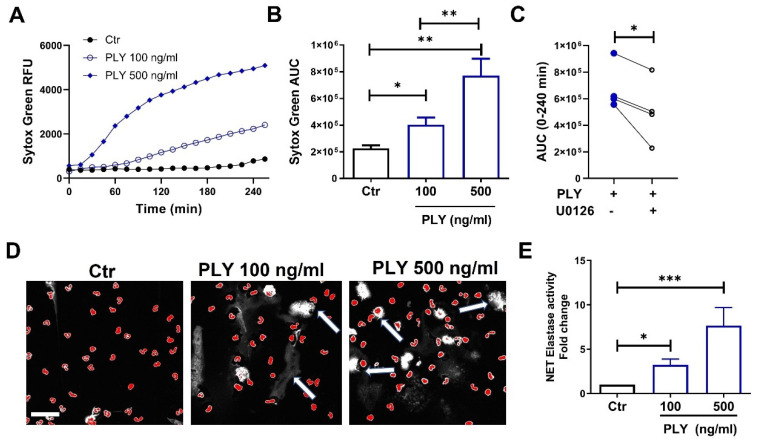
Pneumolysin triggers the formation of neutrophil extracellular traps (NETs). (**A**,**B**) NET formation was quantified using the cell-impermeant nucleic acid dye, SYTOX Green, in human neutrophils treated with PLY (100 or 500 ng/mL). (**A**) NET production (SYTOX Green fluorescence) was monitored over a period of 4 h (representative graph), (**B**) Bars represent the area under the curve (AUC) of Sytox Green fluorescence for Ctr and PLY-treated cells calculated from the kinetic curves. *n* = 12, * *p* < 0.05, ** *p* < 0.01, one-way ANOVA. (**C**) NET production in neutrophils pre-treated with U0126 (25 μM) and then challenged with PLY (100 ng/mL). AUC for each condition was calculated from the kinetic curves (Sytox Green assay). Each line represents neutrophils from the same donor differentially treated. *n* = 4. * *p* < 0.05, paired *t*-test. (**D**) Neutrophils were treated with PLY, fixed, and stained with the nuclear stain DAPI. Depicted are representative pseudocolor images. Red represents intact nuclei. White staining indicates extracellular DNA. Arrows indicate extracellular DNA structures. Pictures were taken at 40x magnification. Scale bar: 50 μm. *n* = 3. (**E**) Elastase activity was measured in isolated NETs from PLY-treated or control neutrophils. *n* = 7–9. * *p* < 0.05, *** *p* < 0.001, one-way ANOVA.

Next, we aimed to investigate whether PLY triggers the production of NETs, as has been suggested recently [42]. To assess the NET formation in PLY-treated neutrophils, we employed an established Sytox Green kinetic assay that allows the monitoring of DNA release over time [43]. As depicted in Figure 2A, PLY induces NETosis in a dose- and time-dependent manner. The total DNA release over 4 h (calculated as area under the curve) was increased significantly by 1.77- and 3.4-fold in neutrophils after stimulation with 100 and 500 ng/mL of PLY, respectively (Figure 2B). Previous studies have demonstrated a requirement for the ERK pathway activation in NET formation [44]. To assess whether PLY-induced NETosis is an ERK-dependent process, neutrophils were pre-treated with an ERK signaling inhibitor (MEK inhibitor; U0126) stimulated with PLY, and DNA release was monitored using the Sytox Green assay. As seen in Figure 2C, the pre-treatment of cells with U0126 resulted in a significant reduction in the PLY-induced DNA release. To visualize NET formation, we employed immunofluorescence microscopy and demonstrated increased extracellular DNA staining (white areas indicated by arrows) that resembles NET structures in PLY-treated neutrophils (Figure 2D). Neutrophil elastase is a NET component, and Figure 2E demonstrates that isolated NETs from PLY-activated cells exhibit increased neutrophil elastase activity. 

### 3.3. Pneumolysin Causes the Release of Extracellular Vesicles from Neutrophils

**Figure 3:**
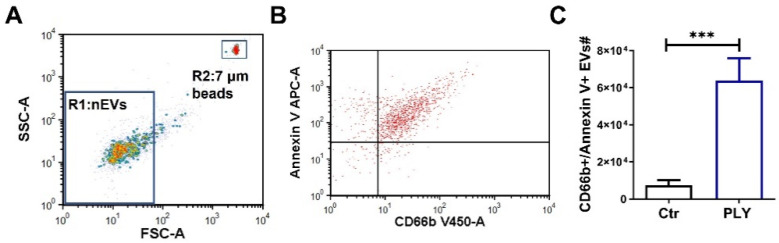
Pneumolysin induces extracellular vesicle release from human neutrophils. Human neutrophils were treated with PLY (100 ng/mL, 1h) or vehicle (Ctr). Neutrophil-EVs (nEVs) were isolated from the cell culture supernatant and analyzed by flow cytometry. (**A**) Representative dot plot of isolated human nEVs. Gate 1 (R1) includes events less than 1 μm; 7 μm counting beads were included in each sample for nEV quantification (gate 2; R2). (**B**) Representative dot plot showing nEVs after staining with annexin V and CD66b (double-positive events are within the upper right quadrant), and (**C**) Quantification of nEVs released from Ctr and PLY-treated neutrophils. *n* = 9. *** *p* < 0.001, paired *t*-test.

We recently demonstrated that PLY is a potent stimulus for EV production from lung epithelial cells [7]. Here, we aimed to explore whether PLY can also stimulate neutrophils to produce EVs. For EV isolation, we employed a standardized protocol that results in the enrichment of larger vesicles (i.e., microvesicles) [7,45]. EVs were isolated from the supernatant of treated human neutrophils and analyzed by FACS, as we and others have described before [7,35,45,46]. As depicted in Supplementary Figure S1A,B, the isolated EVs are less than 1 μm (R1 is a gate that includes beads of 1 μm) and are annexin V positive (annexin V binds to phosphatidylserine (PS) on the outer membrane of EVs). Annexin V staining specificity was determined by staining EVs in the absence of calcium after adding EDTA, a calcium chelator (annexin V binding to PS requires calcium). To further assess their vesicular nature, annexin-V-stained EVs were incubated with the detergent Triton X-100. This is a common assay to discriminate EVs from artifacts, such as debris and immune complexes [7,35,36]. As shown in Supplementary Figure S1C, Triton X-100 completely disrupted and eliminated these EVs. EVs were then defined as events less than 1 μm (Figure 3A) and double positive for annexin V and CD66b (neutrophil marker) (Figure 3B). Under control conditions, neutrophil EV (nEV) levels were low but were significantly increased (by 8.5-fold) after PLY treatment (100 ng/mL, 1 h) (Figure 3C). 

### 3.4. NET and EV Production in Pneumolysin-Treated Murine Neutrophils

**Figure 4:**
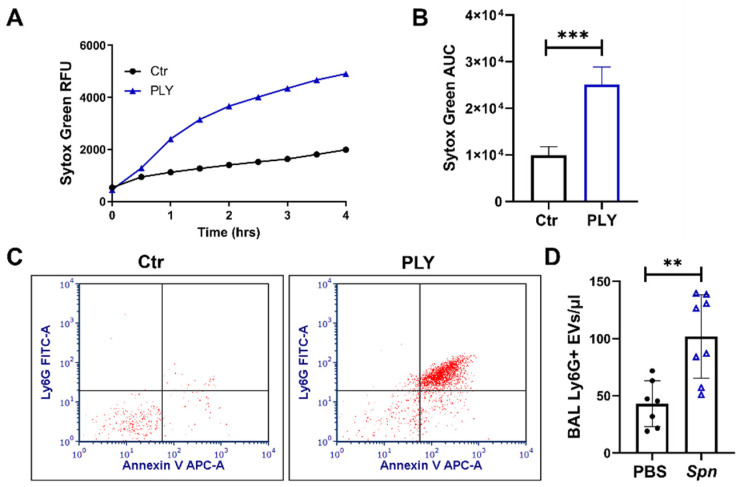
Neutrophil extracellular trap and EV production in murine neutrophils. (**A**,**B**) NET formation was quantified using the cell-impermeant nucleic acid dye Sytox Green in murine neutrophils treated with PLY (100 ng/mL) or HBSS (Ctr). (**A**) NET production (Sytox Green fluorescence) was monitored over a period of 4 h (representative graph). (**B**) Bars represent the area under the curve (AUC) for Ctr and PLY-treated neutrophils calculated from the kinetic curves. *n* = 9, *** *p* < 0.001, paired *t*-test. (**C**) Representative dot blots of nEVs isolated from murine neutrophils treated with vehicle (Ctr) or PLY (100 ng/mL) and stained with annexin V-APC and Ly6G-FITC. Double-positive EVs are within the upper right quadrant. *n* = 3. (**D**) Quantification of nEVs (Ly6G+) in the BAL of mice 48 h after *S. pneumoniae* (*Spn*) infection. *n* = 7–8 mice/group. ** *p* < 0.01, *t*-test.

We next examined the effects of PLY in murine neutrophils. Using the Sytox Green assay, we observed that similar to human cells, PLY causes a significant release of extracellular DNA, indicative of NETosis, from murine neutrophils (Figure 4A,B). Moreover, compared to untreated murine neutrophils, PLY-treated cells release higher amounts of EVs that are smaller than 1 μm and double positive for annexin V and Ly6G (murine neutrophil marker) (Figure 4C). To assess whether nEV levels are increased in vivo, we employed an established and clinically relevant model of S. pneumoniae-induced acute lung injury in mice [37,38,47]. As expected, pneumococcal infection caused a significant accumulation of neutrophils into the lungs (Supplementary Figure S2A). Moreover, BAL Ly6G+EV numbers are elevated by 2.4-fold in mice infected with S. pneumoniae compared to sham-treated (Figure 4D). EVs in BAL were characterized by FACS and defined as events less than 1 μm and annexin V positive (Supplementary Figure S2B,C). 

### 3.5. Effects of Pneumolysin on Platelets

**Figure 5:**
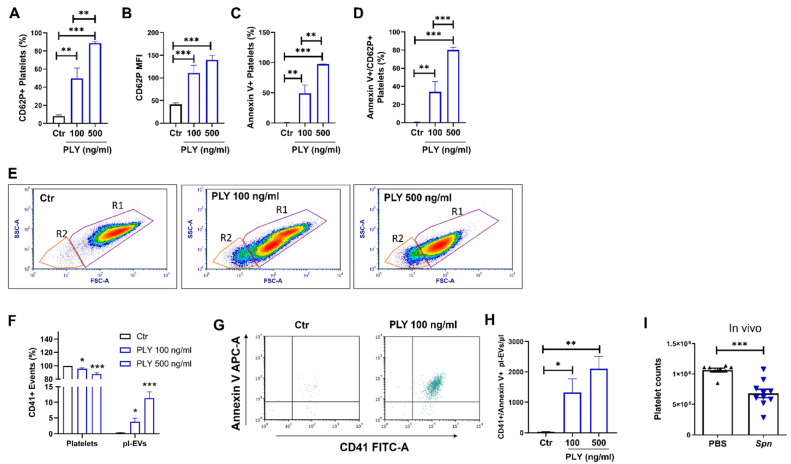
Pneumolysin activates platelets and induces the release extracellular vesicles (pl-Evs). (**A**–**H**) Human platelets (diluted PRP) were stimulated with PLY (100 or 500 ng/mL, 30 min), stained with CD62P (PerCP) antibody or annexin V (APC), and then analyzed by flow cytometry. (**A**) Percentage of CD62P positive platelets, (**B**) Median fluorescence intensity (MFI) of CD62P, (**C**) Percentage of annexin V positive platelets, and (**D**) Percentage of CD62P/annexin V double-positive platelets. (**E**) Representative FSC versus SSC dot plots of resting platelets (Ctr) or platelets treated with PLY (100 or 500 ng/mL). R1 gate includes platelets and R2 gate includes pl-EVs. (**F**) Quantification of the percentage of platelets and pl-EVs for each condition. (**G**) Representative dot plots of pl-EVs (gate R2; (**E**)) and (**H**) corresponding quantification of EVs from Ctr or PLY-treated platelets (CD41/annexin V double positive). (**I**) Platelet counts in whole blood of mice infected with *S. pneumoniae* (*Spn*) (48 h). * *p* < 0.05, ** *p* < 0.01, *** *p* < 0.001. (**A**–**H**) *n* = 7–9, one-way ANOVA, (**F**) comparisons are vs. corresponding controls, (**I**) *n* = 8–10 mice per condition, Mann–Whitney test.

Platelets and platelet–neutrophil interactions play important roles in pneumococcal pneumonia pathogenesis, however, the mechanisms by which pneumolysin can affect platelets are incompletely understood. To address this, we employed flow cytometry to analyze platelets after PLY stimulation. Platelets were identified based on size (forward scatter; FSC vs. Side scatter; SSC, see also gate R1, Figure 5E) and CD41 staining (platelet marker). To assess platelet α-granule secretion, we determined the percentage and expression (MFI) of cell-surface P-selectin (CD62P). Within 30 min of stimulation with PLY, 50 and 89% of platelets were positive for CD62P at doses of 100 and 500 ng/mL of toxin respectively (Figure 5A). Similarly, the expression of CD62P (MFI) was also significantly increased in PLY-treated platelets in a dose-dependent manner (Figure 5B). Activated platelets expose phosphatidylserine (PS) at their surface, promoting the coagulation process [48]. As shown in Figure 5C, PLY at a dose of 100 ng/mL caused a significant increase in the percentage of annexin V positive cells (annexin V binds PS), while almost all platelets treated with 500 ng/mL of PLY were annexin V positive. As expected, the percentage of annexin V/CD62P double-positive platelets was also increased significantly after PLY treatment in a dose-response manner (Figure 5D).

Platelet activation results in the formation of distinct subpopulations that can be detected by flow cytometry [49]. Examining platelets in the FSC vs. SSC plots, we found that unstimulated platelets appear as a single population (gate R1). Treatment with the lower dose of PLY (100 ng/mL) generated a subpopulation, located at the lower FSC–SSC region (gate R2) consisting of very small size events (<1 μm), which corresponds to EVs derived from platelets (pl-EVs) (Figure 5E). Within gate R1, after stimulation with PLY (100 ng/mL), we also observed the formation of an additional subpopulation of platelets that appear smaller in size (Figure 5E). CD62P and annexin V staining were particularly increased in the smaller platelets (data not shown), suggesting that this subpopulation consists of highly activated platelets. Treatment with the higher dose of PLY (500 ng/mL) further increased the number of events in the R2 gate (pl-EVs), while all platelets (within gate R1) had a smaller size (Figure 5E). The percentage of events for each gate (R1: platelets, R2: pl-EVs) were calculated for each condition and are presented in Figure 5F. Platelet-derived EVs are positive for CD41 and annexin V, as seen in Figure 5G. Under resting conditions, pl-EV levels are low but increase significantly after treatment with PLY (Figure 5G,H). Increased production of EVs by PLY was also observed in murine platelets (Supplementary Figure S3). Interestingly, in mice, 48 h after infection with *S. pneumoniae*, platelet counts were significantly decreased by 36% compared to sham-infected mice (Figure 5I). 

### 3.6. PLY-Induced Neutrophil Extracellular Vesicles but Not NETs Activate Platelets 

**Figure 6:**
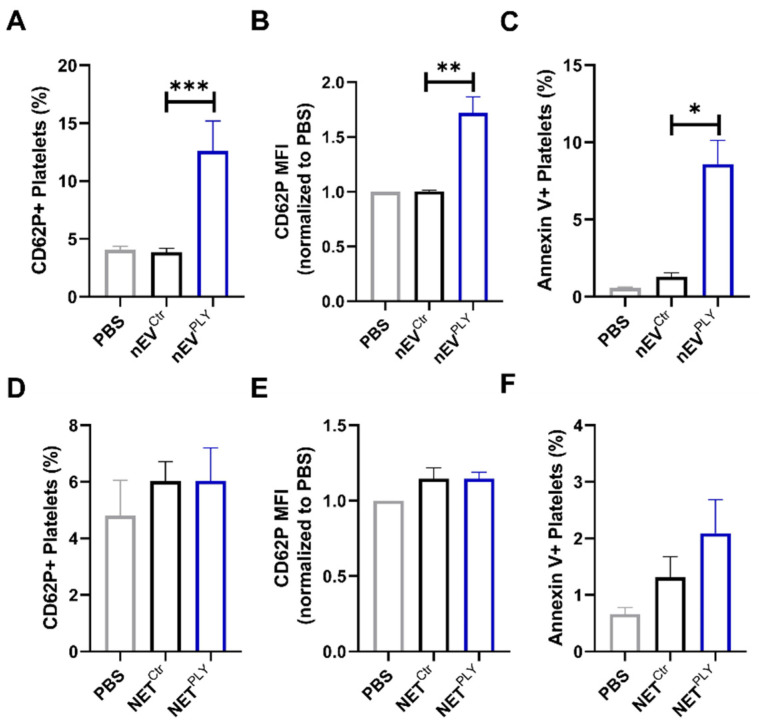
Effects of neutrophil extracellular traps (NETs) and vesicles (nEVs) on platelets. Human platelets were stimulated with nEVs (A–C) or NETs (D–F) from control or PLY-stimulated neutrophils, and CD62P/annexin V expression was assessed by flow cytometry. (**A**) Percentage of CD62P positive platelets, (**B**) median fluorescence intensity (MFI) of CD62P in platelets, and (**C**) percentage of annexin V positive platelets treated with nEVs (derived from Ctr- or PLY-treated neutrophils; nEV^Ctr^, nEV^PLY^). (**D**) Percentage of CD62P positive platelets, (**E**) MFI of CD62P in platelets, and (**F**) percentage of annexin V positive platelets treated with NETs (from Ctr- or PLY-treated neutrophils; NET^Ctr^, NET^PLY^). *n* = 5–10. * *p* < 0.05, ** *p* < 0.01, *** *p* < 0.001, Kruskal–Wallis test.

Recent studies have shown that neutrophil extracellular products, such as NETs derived from PMA-activated neutrophils, contribute to platelet activation [28]. To investigate the potential mechanisms by which PLY could stimulate neutrophil–platelet interaction, we investigated the effects of PLY-induced neutrophil extracellular products (NETs and nEVs) on platelets. For this, nEVs or cell-free NETs isolated from control or PLY-stimulated human neutrophils were added to naïve platelets, which were then analyzed by FACS. As depicted in Figure 6A,B, nEVs from PLY-treated cells (nEV^PLY^) caused significant increases in both the percentage of platelets expressing CD62P, as well as the CD62P surface expression compared to nEVs from Ctr cells (nEV^Ctr^). Treatment with nEV^PLY^ also enhanced the percentage of platelets positive for annexin V compared to nEV^Ctr^ (Figure 6C). On the contrary, we did not observe a consistent effect of NETs from PLY-treated neutrophils (NET^PLY^) on platelet activation, assessed by CD62P cell surface expression and annexin V binding levels (Figure 6D–F). Furthermore, the stimulation of platelets with EV-depleted supernatants (cell culture media from Ctr- or PLY-treated neutrophils collected after nEV isolation) did not cause platelet activation (Supplementary Figure S4). 

**Figure 7:**
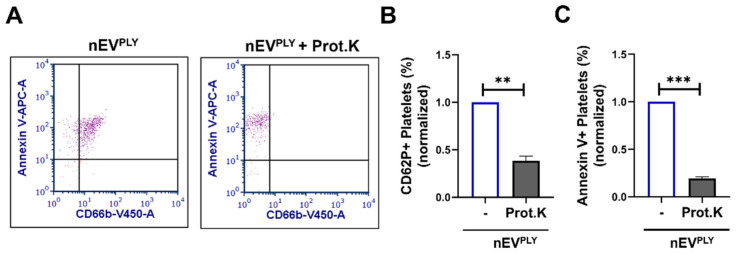
Neutrophil extracellular vesicle (nEV) surface proteins mediate platelet activation. Human platelets were stimulated with nEVs (pre-treated with proteinase K or not) from PLY-stimulated neutrophils (nEV^PLY^). nEVs and platelets were analyzed by flow cytometry. (**A**) Staining of nEV^PLY^ with CD66b (Pacific Blue) and annexin V (APC) shows that proteinase K treatment effectively removes proteins from EVs but not lipids (annexin V binds to PS). Representative dot plots are depicted. Percentage of (**B**) CD62P positive or (**C**) annexin V positive platelets treated with nEV^PLY^ (proteinase K pre-treated or not). *n* = 3. ** *p* < 0.01, *** *p* < 0.001, Welch’s *t*-test.

To further understand the interaction between nEVs and platelets, we incubated nEV^PLY^ with proteinase K, which digests the surface-exposed proteins of EVs [50,51,52]. As shown in Figure 7A, proteinase K treatment successfully removed CD66b from the surface of nEVs and did not affect annexin V binding to phosphatidylserine of EVs. Importantly, the stimulation of platelets pretreated with proteinase K with nEV^PLY^ resulted in decreased percentage of CD62P positive platelets compared to non-proteinase K-nEV^PLY^-treated platelets (Figure 7B). The percentage of annexin V positive cells was also significantly reduced after treatment with nEV^PLY^/Proteinase K (Figure 7C). These data suggest that the nEV surface proteins mediate the interaction of nEVs with platelets.

## 4. Discussion

In this study, we have demonstrated that pneumolysin activates human and murine neutrophils to produce NETs and triggers the release of extracellular vesicles from both neutrophils and platelets (human and murine). We have also provided evidence that neutrophil EVs from PLY-treated cells cause platelet activation. This interaction between neutrophils and platelets through EVs represents a new mechanism by which these cell types interact upon PLY stimulation, and therefore, our studies contribute to a better understanding underlying this toxin’s effect on the host. PLY’s roles as key virulence factor of *S. pneumoniae* and critical mediator of pneumococcal disease pathogenesis have long been recognized [4,6]. Furthermore, PLY’s contribution to the development of complications during *S. pneumoniae* infections, including ARDS, sepsis, and myocarditis, is well-established [53,54]. Therefore, a better understanding of the mechanisms by which PLY injures the host could undoubtedly provide benefits for a wide range of pathologic conditions. At a cellular level, PLY is released from *S. pneumoniae* and mediates its injurious effects primarily by forming pores to membranes on the target cells leading to cell lysis or downstream signaling due to calcium influx or by activating membrane receptors [6]. The concentration of PLY is an important factor in determining its effect on the host. For instance, high concentrations of PLY (2 μg/mL) cause significant plasmalemmal perforations and cell lysis [55]. For our studies, we tested the effects of PLY using sublytic and clinically relevant concentrations of 100 and 500 ng/mL. In vitro experiments have shown that PLY concentration in the bacterial culture supernatant ranges from 200 to 800 ng/mL [55]. In vivo, PLY concentrations up to 180 ng/mL have been detected in cerebrospinal fluid of meningitis patients [56].

Neutrophils are in the first line of defense against bacteria, and although they are required for *S. pneumoniae* clearance, their excessive activation and recruitment are associated with host damage, the aggravation of pneumonia, and the induction of lung injury [9]. This can be partially attributed to the interactions that occur between PLY and neutrophils, which lead to neutrophil dysfunction and compromised immune responses. Here, we demonstrated that neutrophils in response to PLY become activated as assessed by increased CD11b expression and CD62L (L-selectin) downregulation (Figure 1A,B). CD11b regulates leukocyte adhesion and migration to mediate inflammatory responses, while CD62L is an adhesion molecule that mediates neutrophil rolling and sheds upon neutrophil activation. In addition, and consistent with previous studies [57,58,59,60], we observed that PLY caused an increased release of elastase, suggestive of degranulation and enhanced ROS production (Figure 1C,D). A recent study demonstrated that PLY activates NET formation [42]. Our findings support this observation and further demonstrate that the inhibition of the MEK-ERK signaling decreased PLY-induced NETs (Figure 2). The Raf-MEK-ERK signaling pathway is involved in NET formation through NADPH oxidase and the upregulation of anti-apoptotic proteins in PMA-treated neutrophils [44], and our data suggest that PLY-induced NET formation is at least partially dependent on this ERK pathway. Upon their formation, the primary role of NETs is antimicrobial, however, numerous studies to date have demonstrated that they can also cause deleterious effects to the host. For example, NETs cause lung endothelial barrier disruption [61] and promote coagulation and thrombosis [62]. Therefore, it is not surprising that increased NET production has been linked with several pathophysiologic processes, including pneumonia, sepsis, and ARDS [12,63,64,65], and that NETs represent promising therapeutic targets.

Upon activation, neutrophils release EVs that carry cargo to target cells facilitating inter-cellular communications [66]. For example, miR-223 is transferred from fMLP-activated neutrophils to alveolar epithelial cells through nEVs [67], while arachidonic acid within nEVs is taken up by platelets to enhance thromboxane production [29]. Here, we demonstrated that PLY results in significant generation of EVs from both human and murine neutrophils (Figure 3 and Figure 4C). This is consistent with previous work from us and others reporting that PLY triggers EV shedding from lung epithelial cells [7], HEK-293 [68], U937, and THP-1 [69]. Moreover, we found increased levels of nEVs in the alveolar space of mice with lung injury caused by live *S. pneumoniae* (Figure 4D). Interestingly, although there was a ~50-fold increase in neutrophil counts upon infection (Supplementary Figure S2A), nEVs were only increased by 2.4-fold. One possible explanation is that nEVs are rapidly consumed upon production to activate other cells. Nevertheless, these findings are consistent with previous in vivo studies, which showed that intra-alveolar nEVs are associated with lung injury after LPS or mechanical ventilation [35,67], models of lung injury that are characterized by increased neutrophil infiltration into the lungs comparable with *S. pneumoniae* infection. Findings from human studies have also suggested that nEVs are elevated in patients with ARDS, sepsis, and pneumonia (reviewed in [70]). Taken together, our current findings contribute to the prior work suggesting that EVs derived from neutrophils are elevated in conditions associated with lung infection and injury and support the hypothesis that nEVs are also involved in *S. pneumoniae* infections. Although we did not assess the role of PLY in the production of nEVs in vivo in this current study, our prior work has demonstrated that PLY represents the major *S. pneumoniae* virulence factor responsible for EV release from epithelial cells [7]. Specifically, in that prior study, we demonstrated that a PLY deficient *S. pneumoniae* strain failed to induce EV release from epithelial cells in vitro and ex vivo. Therefore, it is reasonable to speculate that nEV production in vivo upon *S. pneumoniae* infection is primarily dependent on PLY expression. However, additional studies are needed to explore the specific mechanisms of nEV production in vivo after pneumococcal infection.

In addition to stimulating neutrophil activation and EV release, we found that PLY also induces platelet EV generation (Figure 5F–H). Similar to other cells, EVs are produced by platelets upon activation and have been associated with numerous diseases, including pneumonia caused by different pathogens (e.g., SARS-CoV-2) [24,25]. EVs derived from platelets represent the primary source of EVs in circulation, and while earlier studies focused on their potent procoagulant activities, it is becoming evident that they have diverse functions [24]. Calcium influx is one of the most potent signals for EV release, and since PLY functions as a calcium ionophore, it is not surprising that it can trigger EV release from platelets, similar to other cell types. Comparable with PLY, other bacterial toxins, such as the staphylococcal superantigen-like protein 5 (SSL5), can activate platelets to produce EVs, which then bind to monocytes to induce aggregation and release of pro-inflammatory cytokines [71].

PLY-induced EV release was associated with platelet activation indices, such as increased CD62P (P-selectin) expression and annexin V positivity (Figure 5A–D). P-selectin is stored in α-granules in platelets and translocates to the cell surface upon activation. PLY-induced CD62P expression and degranulation upon PLY or *S. pneumoniae* treatment has been reported previously [72,73,74]. A recent study suggested that the increased CD62P expression in PLY-treated platelets is not induced by activation but is caused by the destruction of platelets and elevated intracellular staining due to increased pore formation [75]. Previous reports, however, have also shown that PLY causes neutrophil–platelet complex formation [76] (observations that we also confirmed in our experimental settings (data not shown)), which is mediated by P-selectin (exposed on the surface of activated platelets) and its ligand PSGL-1 on neutrophils [77]. This strongly indicates that PLY mobilizes CD62P on the platelet membrane leading to their attachment with neutrophils. Moreover, in our study, upon PLY stimulation, we observed by FACS (Figure 5E) the formulation of three distinct platelet subpopulations (normal size platelets, small size platelets, and platelet EVs), a pattern that has previously been suggested as an indicator of potent platelet activation [49]. Specifically, increasing concentrations of PLY led to an increase in the percentage of smaller size platelets and pl-EVs (Figure 5E,F). In vivo, we observed a significant reduction in circulating platelet counts in mice after infection with *S. pneumoniae* (Figure 5I). Decreased platelet numbers in association with platelet activation has also been reported in murine sepsis induced by *S. pyogenes* [78]. In patients, the over-activation of platelets and the reduction in platelet counts have also been correlated with the severity of pneumonia, ARDS, and sepsis [79,80]. 

Although neutrophils and platelets have their individual critical roles in pneumonia, it is likely that their interactions are also important. There are different mechanisms by which neutrophil–platelet interactions drive inflammatory processes that include the formation of neutrophil–platelet complexes and crosstalk through direct and indirect contact [26]. Platelets are known to contribute to NET production [27,81], while PMA-induced NETs promote platelet activation [28]. In our study, PLY-induced NETs had no significant effect on platelet activation (Figure 6D–F). It is possible that different stimuli result in the production of NETs with distinct compositions that also define their functional roles. Other factors, such as the specific experimental conditions used in the different studies (for example, NET preparations or incubation times), may have impacted the outcome on platelet activation. We propose that additional studies should be performed to clarify the role of NETs in platelet functions. In contrast to the negative effects of NETs in our study, nEVs from PLY-stimulated neutrophils caused a moderate but significant activation of platelets as demonstrated by the induction of platelet CD62P expression and annexin V binding (Figure 6A–C). This is consistent with some previous studies that examined the interactions between nEVs and platelets upon other stimuli. PMA-triggered nEVs were found to bind to platelets through αMβ2 and PSGL-1 receptors and induce platelet CD62P [30]. In another study, EVs from ADP/fMLP-treated neutrophils enhanced thromboxane production from activated platelets, an interaction that was critical for the immune response to *E. coli* lung infection [29]. 

EVs’ specific biological functions are dependent on their distinct molecular cargo, which is defined by the EV cellular origin and the triggering stimulus. In this study, we focused on the effects of PLY-induced nEVs on platelet activation. Since PLY induces EV release from multiple cell types, including lung epithelial cells, as we recently showed [7], it would be interesting to explore whether EVs derived from PLY-stimulated cells other than neutrophils could also activate platelets. However, it is likely that the cargo carried by PLY-induced EVs will differ based upon the cellular type of origin and therefore exhibit differential functional properties. In addition, previous studies have reported that PLY itself can be incorporated into the EV cargo [55,68]. Given that PLY activates platelets, its presence within EVs could explain their functional role. In preliminary studies, we assessed PLY content in PLY-induced nEVs by Western blotting, however, it was not detected (data not shown). Moreover, EV-depleted supernatant from PLY-treated neutrophils failed to activate platelets (Supplementary Figure S4). Therefore, even if the supernatant (and by extension the EVs isolated from this supernatant) contain PLY, it must be at a very low level (and/or in an inactive state) since the supernatant has no effect on platelets. Although these data suggest that cargo other than PLY is responsible for platelet activation, additional studies are required to characterize the functional effectors within these EVs. 

Our data further demonstrated that the EV–platelet interaction was primarily mediated by nEV surface proteins, as their digestion (after treatment with Proteinase K) resulted in diminished nEV capacity to activate platelets (Figure 7). Previous studies have reported that the digestion of EV surface proteins by proteinase K reduces their uptake from target cells [51]. Whether nEVs cause platelet activation due to their uptake by platelets or due to surface interactions will be the focus of future studies. Finally, although the significance of neutrophil–platelet interactions through PLY-derived EVs remains to be elucidated, it is likely that they contribute to hypercoagulation, as has been suggested by other studies of neutrophils EVs [82].

## 5. Conclusions

In conclusion, our study demonstrates for the first time that EVs derived from pneumolysin-treated neutrophils can directly cause platelet activation. Further studies will be needed to specifically address the mechanisms by which neutrophil EVs may alter platelet function both in vitro and in vivo upon pneumococcal infections and determine the consequences of nEV–platelet interaction in the disease process. 

## Data Availability

The datasets presented in this study are available upon request from the corresponding author.

## Supplementary Materials

The following are available online at https://www.mdpi.com/article/10.3390/cells10123581/s1, Figure S1: Neutrophil-derived extracellular vesicle (nEV) characterization, Figure S2: Bronchoalveolar lavage neutrophils and EVs, Figure S3: Increased production of EVs from murine platelets treated with pneumolysin, Figure S4: Effects of neutrophil supernatant on platelets.
