# Supplementary material for: Neutrophil-Derived Extracellular Vesicles Activate Platelets after Pneumolysin Exposure
Source: Cells. 2021 Dec 18;10(12):3581. doi: 10.3390/cells10123581 (PMC8700313; doi:10.3390/cells10123581)
Supplement: Supplementary file 1 [file cells-10-03581-s001.zip › cells-1470108-supplementary.pdf]

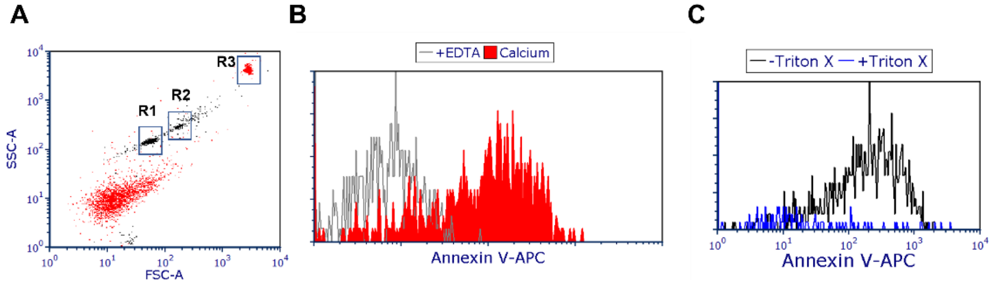

Supplementary Figure S1. Neutrophil-derived extracellular vesicle (nEV) characterization. Human neutrophils were treated with PLY (100 ng/ml) and nEVs were isolated from the cell culture supernatant and characterized by flow cytometry. (A) nEVs are smaller than 1 µm. Representative dot plot of isolated human nEVs and sizing beads that were run in parallel. Gate 1 (R1) includes beads of 1 µm, gate R2 beads of 2 µm and gate R3 beads of 7 µm (counting beads). (B) nEVs are annexin V positive. Representative histogram showing nEVs after staining with annexin V in the presence (red) or absence of calcium (open histogram-no fill), and (C) nEVs are membrane vesicles that can be disrupted by the detergent, Triton X-100. Representative histogram of annexin V-stained nEVs after Triton X-100 treatment.

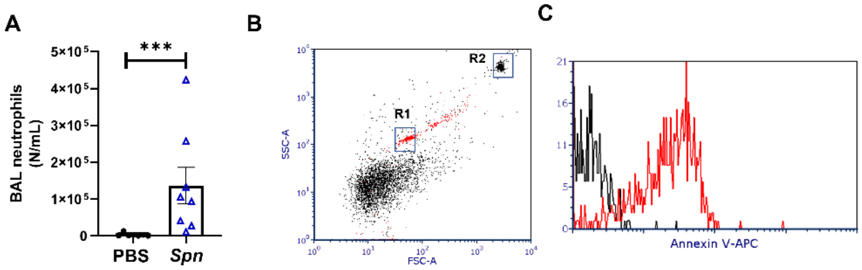

Supplementary Figure S2. Bronchoalveolar lavage neutrophils and EVs. A. Neutrophil counts were determined in BAL of mice 48 hours after *S. pneumoniae* (*Spn*) infection. N=7-8 mice/group. ***p<0.001, Mann-Whitney test. **B.** Representative dot plot of BAL EVs and sizing beads that were run in parallel. Gate 1 (R1) includes beads of 1 µm and gate R2 beads of 7 µm (counting beads). **C.** BAL EVs are annexin V positive. Representative histogram showing BAL EVs after staining with annexin V in the presence (red line) or absence of calcium (black line).

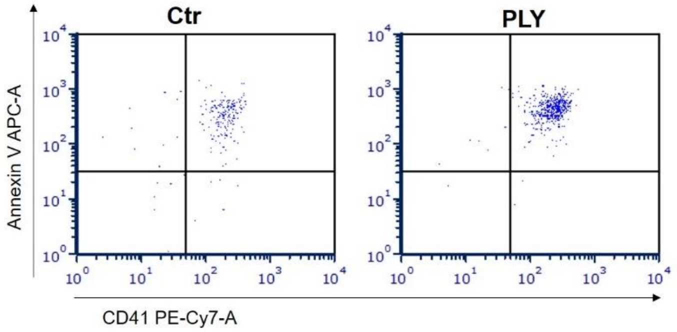

Supplementary Figure S3. Increased production of EVs from murine platelets treated with pneumolysin. Murine platelets were treated with PLY or PBS (control). The platelet suspension was stained with annexin V-APC and CD41-PE-Cy7 and analyzed by FACS. Depicted are representative dot plots of events less than 1 μm that represent the EV population in Ctr and PLY-treated platelets. PLY induces increased production of annexin V/CD41 double positive EVs. N=3.

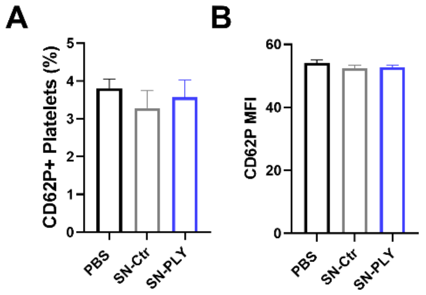

Supplementary Figure S4. Effects of neutrophil supernatant on platelets. Human platelets were stimulated with supernatant, SN (depleted of large EVs) from control or PLY-stimulated neutrophils, and CD62P expression was assessed by flow cytometry. (A) Percentage of CD62P positive platelets, and (B) median fluorescence intensity (MFI) of CD62P in platelets. N=6.
